# Altered glutamyl-aminopeptidase activity and expression in renal neoplasms

**DOI:** 10.1186/1471-2407-14-386

**Published:** 2014-05-30

**Authors:** Lorena Blanco, Begoña Sanz, Itxaro Perez, Clara E Sánchez, M Luz Cándenas, Francisco M Pinto, Javier Gil, Luis Casis, José I López, Gorka Larrinaga

**Affiliations:** 1 Department of Physiology, Faculty of Medicine and Dentistry, Universitiy of the Basque Country (UPV/EHU), Leioa, Bizkaia, Spain; 2 Department of Nursing I, Faculty of Nursing, Universitiy of the Basque Country (UPV/EHU), Leioa, Bizkaia, Spain; 3 Institute for Chemical Research, CSIC, Sevilla, Spain; 4 Department of Anatomic Pathology, Cruces University Hospital, University of the Basque Country (UPV/EHU), Barakaldo, Bizkaia, Spain

**Keywords:** Glutamyl-aminopeptidase, Aminopeptidase A, Angiotensinase, Angiotensin, Clear cell renal cell carcinoma, Renal neoplasm

## Abstract

**Background:**

Advances in the knowledge of renal neoplasms have demonstrated the implication of several proteases in their genesis, growth and dissemination. Glutamyl-aminopeptidase (GAP) (EC. 3.4.11.7) is a zinc metallopeptidase with angiotensinase activity highly expressed in kidney tissues and its expression and activity have been associated wtih tumour development.

**Methods:**

In this prospective study, GAP spectrofluorometric activity and immunohistochemical expression were analysed in clear-cell (CCRCC), papillary (PRCC) and chromophobe (ChRCC) renal cell carcinomas, and in renal oncocytoma (RO). Data obtained in tumour tissue were compared with those from the surrounding uninvolved kidney tissue. In CCRCC, classic pathological parameters such as grade, stage and tumour size were stratified following GAP data and analyzed for 5-year survival.

**Results:**

GAP activity in both the membrane-bound and soluble fractions was sharply decreased and its immunohistochemical expression showed mild staining in the four histological types of renal tumours. Soluble and membrane-bound GAP activities correlated with tumour grade and size in CCRCCs.

**Conclusions:**

This study suggests a role for GAP in the neoplastic development of renal tumours and provides additional data for considering the activity and expression of this enzyme of interest in the diagnosis and prognosis of renal neoplasms.

## Background

Clinical data support the fact that renal cell carcinomas (RCCs) are neoplasms with high prevalence and mortality rates [1]. The 2004 WHO classification of renal tumours in adults includes newly reported entities [2] and links the classical histological findings of these neoplasms to a wide spectrum of genetic abnormalities [3] still not fully defined. Clear cell renal cell carcinoma (CCRCC) is by far the most frequent histological subtype, accounting for approximately 70% of the cases. The proximal convoluted tubule is the proposed site of origin for CCRCC. Papillary renal cell carcinoma (PRCC) is the second most frequent subtype (10%–15%) and also arises in the proximal convoluted tubule. Chromophobe renal cell carcinoma (ChRCC) and renal oncocytoma (RO), both of which originate from the intercalated cells of the collecting ducts in the distal nephron and are thought to share a common lineage, are much less frequent, accounting for approximately 5% of the cases each [2].

At present, there is no clinical marker to detect the disease in the asymptomatic potentially curable phase or to reliably predict the clinical course of every case. Only classic pathological parameters such as histological subtype, tumour stage and grade may contribute to that purpose. However, depending on the clinical setting and other circumstances, many renal tumours escape the expected behaviour and this means it is necessary to discover more predictable parameters [4].

Recent findings have revealed the implication of several proteases in the genesis, growth and dissemination of renal neoplasms. Significant effort has been made towards understanding the role of matrix metalloproteinases [4-7]. In parallel, an increasing number of studies show significant changes in the expression and activity of peptidases in these tumours and point to these proteins not only as potential diagnostic and prognostic markers but also as therapeutic targets [8-12].

Glutamyl-aminopeptidase (GAP), also known as aminopeptidase A (EC. 3.4.11.7), is a membrane-bound zinc metallopeptidase that removes N-terminal acidic residues from peptides such as angiotenin II, its best known natural substrate [13]. GAP has a widespread tissue distribution and participates in many diverse biological processes [14]. This enzyme is involved in the development of kidney structures [14] and is expressed in the glomerulus and in the tubular system of the nephron [15], where it has been described as the gp160 human kidney differentiation antigen [16].

Immunoassay and semiquantitative enzymatic studies in renal cell carcinoma cell lines and in primary renal cancers have shown altered GAP expression and activity in these neoplasms [8,9,17]. It has also been reported that GAP expression is correlated with resistance to the antiproliferative effect of interferon-α in RCCs [18]. These data suggest that the study of GAP activity and expression may have diagnostic and prognostic applications for clinical practice. To clarify this question we quantified GAP activity and analysed its immunohistochemical expression in a wide range of renal neoplasms. We selected renal tumours with different histogenetic origins and aggressiveness (CCRCC, PRCC, ChRCC and RO), which cover 95% of renal neoplasms. Additionally, the GAP activity profile was compared in different CCRCC grades and stages and was correlated with patient survival.

## Methods

The authors declare that all experiments carried out in this study comply with current Spanish and European Union legal regulations. Samples and data from patients included in this study were provided by the Basque Biobank for Research-OEHUN (http://www.biobancovasco.org). All patients were informed about the potential use for research of their surgically resected tissues, and accepted this eventuality by signing a specific document approved by the Ethical and Scientific Committees of the Basque Country Public Health System (Osakidetza) (CEIC 11/51).

### Tissue specimens

We analysed renal tissue in a series from patients with CCRCC, PRCC, ChRCC and RO. Hospital Ethics Committee approval was obtained a priori. Fresh tissue samples were obtained from surgical specimens from renal tumour patients. Tumour and normal (surrounding uninvolved tissue) areas were obtained in all cases. For activity studies, tissue samples were stored at −80°C until the enzyme assays were performed. In addition, selected tissue samples were formalin-fixed and paraffin-embedded for histopathological studies. The 2004 WHO histological classification of adult renal cell tumours [2] and the 2002 TNM Edition for tumour staging [19] were used for pathological diagnosis. In addition, Furhman’s method [20] was applied for grading CCRCC subtype. Clinical follow up of CCRCC patients was closed by December 31, 2012, and a total of 14 patients died of disease at that time. Mean follow up was 65.2 months (range, 8–130 months).

### Sample preparation

Although GAP has been classically reported as a cell-surface peptidase [16], several studies have also demonstrated GAP activity in soluble fractions of different tissues and cells [21-23]. We therefore analysed GAP activity in both membrane-bound and soluble fractions of kidney tissues.

Explanted tissue samples were homogenised in 10 mM Tris–HCl buffer at pH 7.4, for 30 seconds at 800 rpm using a Heidolph PZR 50 Selecta homogeniser, and ultracentrifuged in a Centrikon T-2070 Kontron Instruments apparatus at 100,000 *g* for 35 min. The resulting supernatants were used to measure cytosolic (soluble) enzyme activities and protein concentrations. To avoid contamination with soluble enzymes, the resulting pellets were washed three times by suspension in 10 mM Tris–HCl buffer at pH 7.4. Pellets were then homogenised in 10 mM Tris–HCl buffer at pH 7.4, and centrifuged at low speed (1500 *g*) for 3 min to purify the samples. The supernatants thus obtained were used to determine membrane-bound enzyme activities and protein concentrations. All the aforementioned steps were carried out at 4°C.

### Measurement of glutamyl-aminopeptidase activity

The enzyme activity of tumour and non-tumour tissue samples from 50 patients with CCRCC (40 men, 10 women; mean age: 63 years), 10 patients with ChRCC (5 men, 5 women; mean age: 64 years) and 8 patients with RO (6 men, 2 women; mean age: 67 years) was analysed by spectrofluorometric methods. GAP activity was measured by a modified version of Tobe et al.’s method [24], using Glu-β-naphthylamide (0.125 mM) as a substrate. The assay is based on the fluorescence of β-naphthylamine generated from the substrate by GAP. The components of the assay mixture (total volume 2 mL) included the following: 50 mM of Tris–HCl buffer (pH 7.4) and 0.15 mg/mL of bovine serum albumin. The reaction was initiated by adding 30 μL of sample to 1 mL of the assay mixture. This was incubated at 37°C for 30 min and the reaction was discontinued by the addition of 1 mL of 0.1 M sodium acetate buffer (pH 4.2). The excitation and emission wavelengths were 345 and 412 nm, respectively. Blanks were used to determine background fluorescence. Relative fluorescence was converted into picomoles of product using a standard curve constructed with increasing concentrations of β-naphthylamine.

Protein concentration was measured in triplicate by the Bradford method [25], using BSA (1 mg/mL) as the calibrator. Results were recorded as units of peptidase (UP) per milligram of protein. One unit is equivalent to the release of one mole of beta-naphthylamine per minute. Fluorogenic assays were linear with respect to hydrolysis time and protein content.

### Immunohistochemistry

Formalin-fixed and paraffin-embedded tumour tissue from 12 CCRCC (9 men and 3 women; mean age: 62 years); 6 PRCC (5 men and 1 woman; mean age: 64 years), 4 ChRCC (2 men, 2 women; mean age: 67 years) and 4 RO (all men, mean age: 63 years) were immunostained with a rabbit polyclonal antibody specific for glutamyl-aminopeptidase (Anti BP-1, Abcam plc, Cambridge, UK, working diluton 1:250). The immunostaining process was performed following routine methods in an automatic immunostainer (Dako Autostainer Plus). In short, endogenous peroxidase activity was blocked by incubating the slides in 3% hydrogen peroxide in absolute methanol for 10 minutes. Antigen retrieval was carried out in citrate buffer (10 mM, pH = 6) for 15 minutes at 100°C in a microwave oven. The primary antibody was applied for 1 hour at room temperature. A subsequent reaction was performed with secondary antibodies and biotin-free HRP enzyme labelled polymer of the EnVision-Flex detection system (Dako, Carpinteria, CA). Nonspecific IgG was used as a negative control. A positive reaction was visualized with diaminobenzydine solution followed by counterstaining with haematoxylin. Two independent observers analysed the immunohistochemical slides separately assigning staining intensity according to a semiquantitative scale [(negative (−), mild (+), moderate (++), and intense (+++)]. Minor disagreements were reconciled under a multihead microscope.

### Quantitation of GAP (*ENPEP*) mRNA expression

Quantitative RT-PCR for detecting *ENPEP* mRNA was performed to assess the transcription levels of this enzyme. The total RNA of tumour tissue samples from 30 CCRCC patients (18 male, 12 female; mean age: 62 years) was isolated following the standard protocol previously described [26].

First-strand cDNA was synthesized from 25 μg of total RNA from each human sample using Moloney murine leukemia virus reverse transcriptase and random hexamers according to the manufacturer’s instructions (first-strand cDNA Synthesis Kit, Amersham Biosciences, Essex, UK). The resulting cDNA samples were amplified by PCR with specific oligonucleotide primer pairs designed with the analysis software Primer 3 [27]. Based on previous experiments on human renal cell carcinoma [28] and other human tissues [29,30], TATA box binding protein (*TBP*), peptidylprolyl isomerase A (*PPIA*), β-actin (*ACTB*) and succinate dehydrogenase complex subunit A (*SDHA*) were chosen as endogenous reference genes. The sequences of the primers used to amplify *ENPEP* and the four housekeeping genes are shown in Table 1. All primers were synthesized and purified by Sigma-Genosys (Cambridge, UK).

**Table 1:** Sequences for forward and reverse primers of the indicated target genes and the size expected for each PCR-amplified product

***Enzyme***	***Gene symbol***	***Forward primer***	***Reverse primer***	***Amplicon size (bp)***
APA	*ENPEP*	5′-GCTCTCCTTGAACCACAAGACA-3′	5′-TTCTCTTCCCTTTTGAGATACTTGG-3′	133
*Housekeeping Gene name*				
β-actin	*ACTB*	5′-TCCCTGGAGAAGAGCTACGA-3′	5′-ATCTGCTGGAAGGTGGACAG-3′	362
Succinate dehydrogenase complex, subunit A	*SDHA*	5′-TCTGCCCACACCAGCACT-3′	5′-CCTCTCCACGACATCCTTCC-3′	142
TATA box binding protein	*TBP*	5′-GGATAAGAGAGCCACGAACCAC-3′	5′-TTAGCTGGAAAACCCAACTTCTG-3′	139
Peptidylpropyl isomerase A	*PPIA*	5′-GGTCCCAAAGACAGCAGAAAA-3′	5′-TCACCACCCTGACACATAAACC-3′	114

Primers for the assayed housekeeping genes are also shown.

Expression of the target and housekeeping genes was quantified in all cDNAs by real-time PCR using the iCycler iQ real-time detection apparatus (BioRad Laboratories, Hercules, CA, USA). Dilutions of the cDNA template were prepared with each tissue and amplified in triplicate using SensiMix Plus SYBR + FLUORESCEIN (Quantace Ltd., London, UK). Three negative controls (with no template, no reverse transcriptase and no RNA in the reverse transcriptase reaction) were also included in each plate to detect any possible contamination. After a hot start (10 min at 94°C), the parameters used for PCR amplification were: 10 s at 94°C, 20 s at 60°C and 30 s at 72°C, for 50 cycles.

Real-time PCR data were expressed as the fold change of the target gene expression relative to the geometric mean (g.m.) mRNA expression of the housekeeping genes in each sample, as described by Vandesompele et al. [31]. The fold change in gene expression was calculated by the formula: $2_{T}^{–∆∆C}$, where C_T_ is the threshold cycle, as calculated by the iCycler software, ∆C_T_ = (C_T_target gene–C_T_g.m.reference genes) and ∆∆C_T_ = (∆C_T_ test sample–∆C_T_ control sample).

CCRCCs with different Fuhrman’s gade and stage (low and high) were always measured in the same analytical run to exclude inter-run variations..

### Statistical analysis

SPSS® 19.0 software (IBM, Madrid, Spain) was used to perform statistical data analysis. A Kolmogorov-Smirnov test was applied to data obtained from tissue to determine whether the numbers followed or not a normal distribution. Based on this information (p < 0.05), data from GAP activity and mRNA levels were analysed with non parametric tests. Mann–Whitney test was used to detect differences between non-tumour and tumour tissues, and between CCRCCs with different Fuhrman’s grade [G1/2 (low) vs. G3/4 (high)] and stage [pT1/2 (organ confined) vs. pT3/4 (non-organ confined)]. Spearman’s correlation (ρ) test was performed to evaluate the correlation between GAP activity, patient age and gender, and tumour size of CCRCC. A value of *P* < 0.05 was considered statistically significant. Finally, overall survival (five years) was assessed by the Kaplan-Meier method and compared by log-rank test according to GAP activity levels.

## Results

### Glutamyl-aminopeptidase activity profile in renal tumours

**Figure 1:**
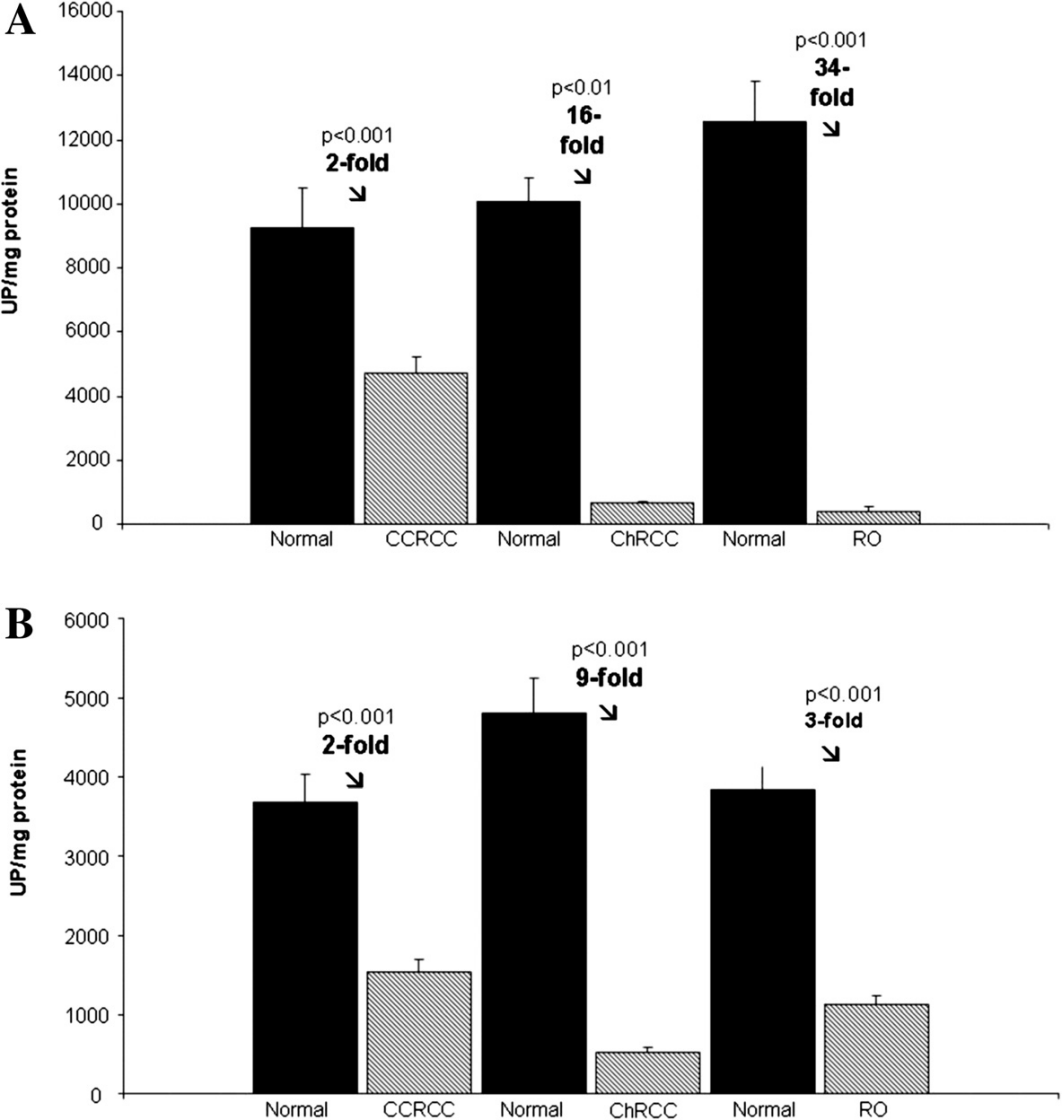
**Glutamyl-aminopeptidase activity profile in the membrane-bound (A) and soluble fraction (B) of CCRCC, ChRCC and RO.** The columns compare tumour with non-tumour surrounding tissue (normal). Values represent mean ± SE of enzyme activities recorded as units of enzyme per milligram of protein (U/mg prot). Mann–Whitney test: (***) *P* < 0.001; (**) *P* < 0.01.

**Figure 2:**
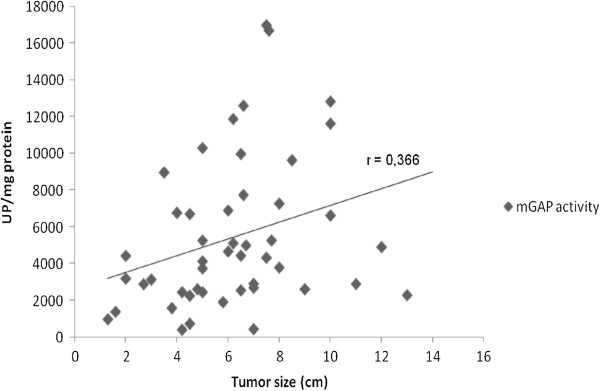
**Representation of the correlation between membrane-bound gultamyl-aminopeptidase and tumour size in CCRCC.** Values represent mean ± SE of enzyme activities recorded as units of enzyme per milligram of protein (U/mg prot). Spearman’s rank test: coefficient (r) = 0.366; *P* < 0.05.

Data obtained in the GAP activity assays across the different tumour types and in stratified CCRCC are reported in Figures 1 and 2.

Figure 1 shows GAP activity measured in tumour and non-tumour tissue (normal) of CCRCC, ChRCC and RO patients. Activity was recorded as pmol of product/min/mg protein (UP/mg protein) and is presented as mean ± SE. As shown in Figure 1A, when compared with non-tumour tissues, membrane-bound GAP activity decreased significantly in CCRCC (two-fold) (Mann–Whitney test, *P* < 0.001), and drastically in ChRCC (16-fold, *P* < 0.01) and RO (34-fold, *P* < 0.001). The soluble GAP activity in renal tumours (Figure 1B) also decreased significantly in all tumour types analysed when compared with normal tissue samples (*P* < 0.001).

Table 2 represents GAP activity in the different grades and stages of CCRCC group (Low grade: G1-G2, n = 24 vs. High grade: G3-G4, n = 26; Low stage: T1-T2, n = 34 vs. High stage: T3-T4, n = 16). Activity was recorded as pmol of product/min/mg protein (UP/mg protein) and is presented as mean ± SE.

**Table 2:** Membrane-bound and soluble GAP activity in CCRCC stratified by grade and stage

			
**a**			
	Low Grade (G1-G2)	High Grade (G3-G4)	*P*
mGAP	6099 ± 1689	6313 ± 1105	ns
sGAP	**1193 ± 231**	**1895 ± 213**	**<0.05**
**b**			
	Low Stage (T1-T2)	High Stage (T3-T4)	*P*
mGAP	6322 ± 1169	5699 ± 893	ns
sGAP	1563 ± 210	1693 ± 310	ns

Values are means ± SE of Units per mg of protein (U/mg Prot). *Abbreviations*: ns not significant. Statistically significant results are in bold.

After stratification by grade, soluble GAP showed higher activity in CCRCCs with a high Furhman’s grade in comparison to those clear cell carcinomas in a lower grade (Mann–Whitney test, *P* < 0.05). Analyses of grading for membrane-bound GAP and staging for both soluble and membrane-bound GAP activities were not statistically significant.

The correlation study demonstrated that membrane-bound GAP activity is positively correlated with tumour size in CCRCC samples (Spearman’s rank test, *P* < 0.05) (Figure 2).

**Figure 3:**
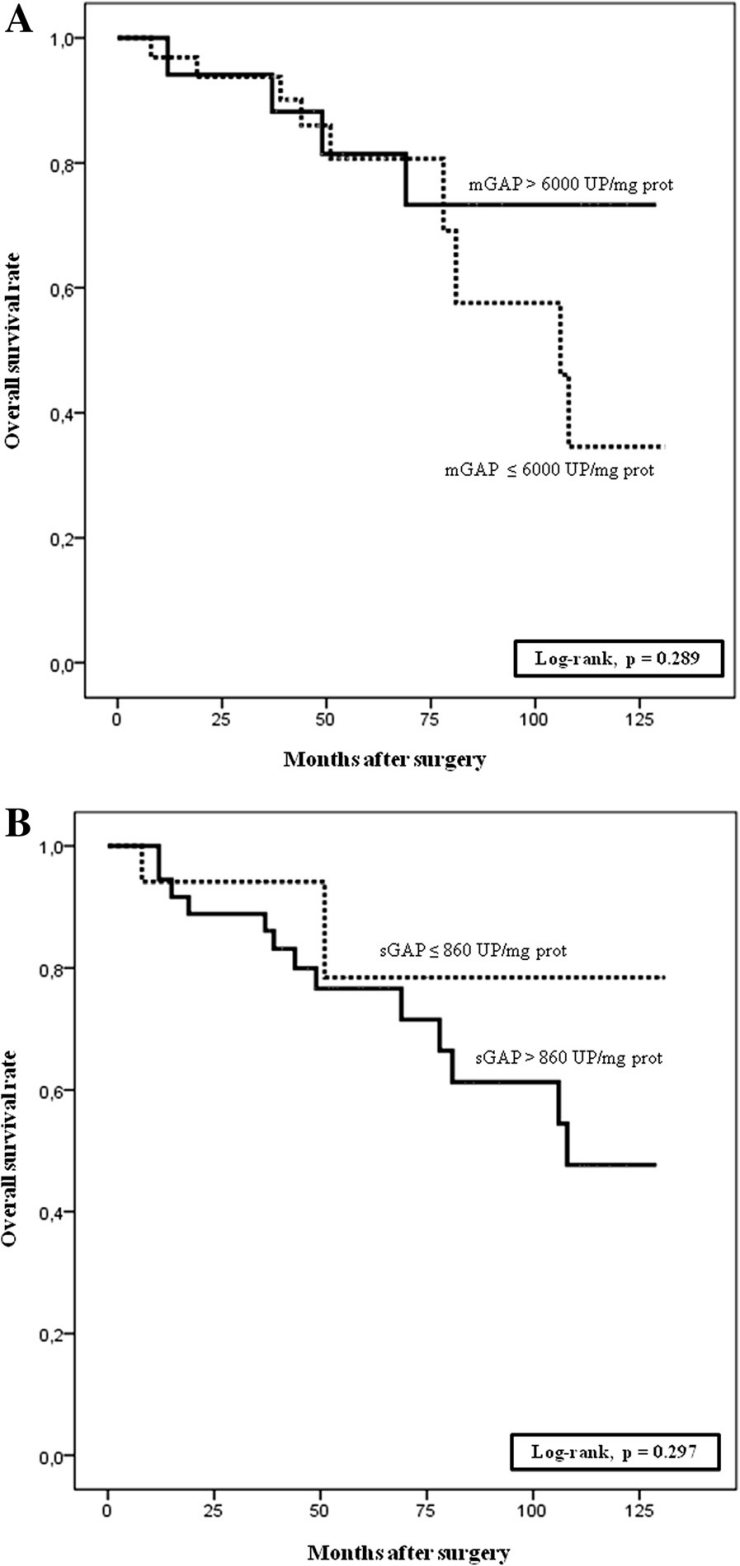
**Patient survival curves according to the membrane-bound (A) and soluble (B) glutamyl-aminopeptidase activity levels (Kaplan-Meier method).** GAP activity did not significantly impact in survival of patients with CCRCC (log-rank p > 0.05).

Finally, Kaplan-Meier curves revealed that GAP activity was not correlated with patients’ five-year survival (log-rank test *P* > 0.05) (Figure 3A and B).

### Glutamyl-aminopeptidase immunohistochemical expression

**Figure 4:**
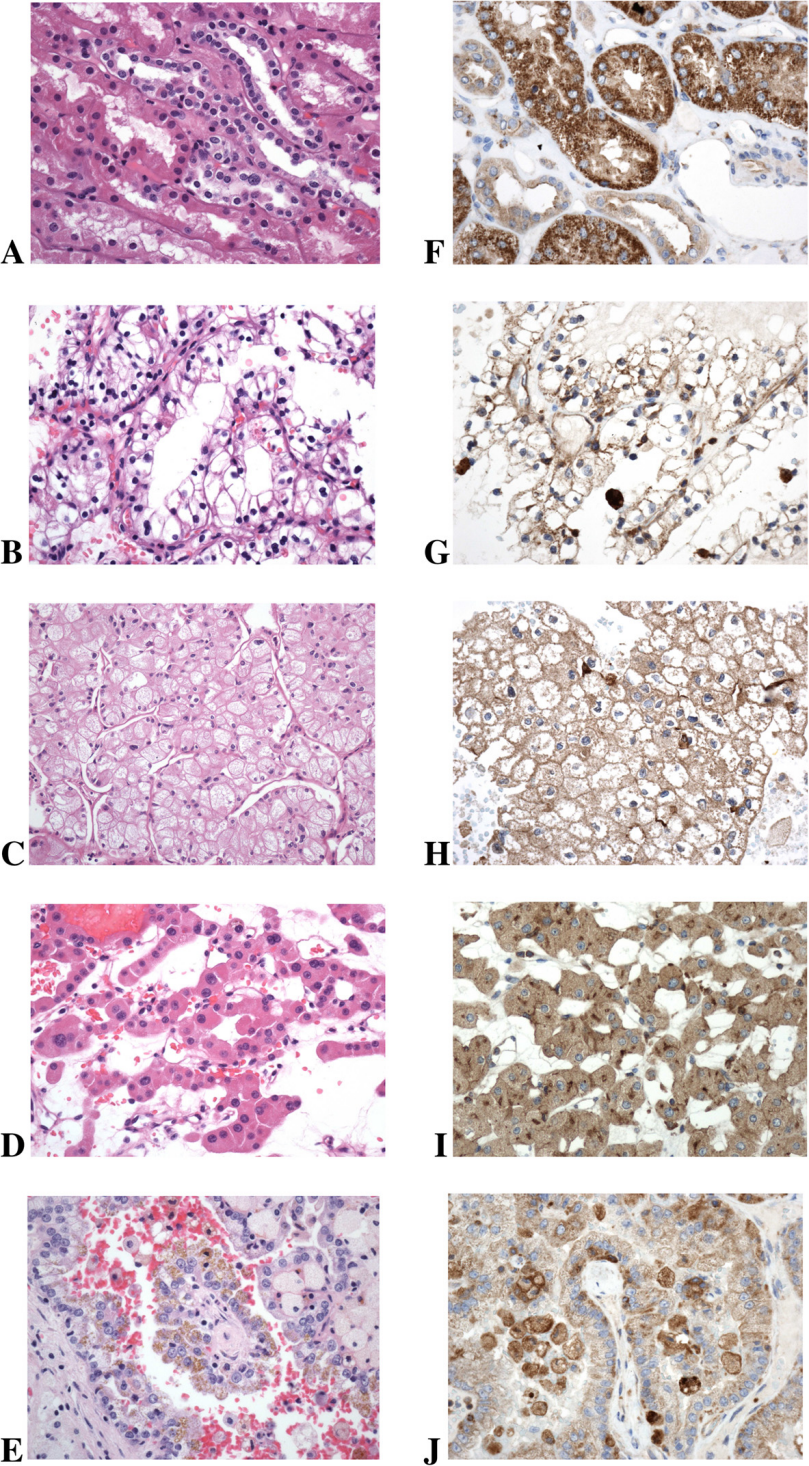
**Immunohistochemistry of GAP in renal tumours.** Haematoxylin and Eosin (HE) staining of **(A)** normal renal tissue, **(B)** clear cell renal cell carcinoma, **(C)** chromophobe renal cell carcinoma, **(D)** renal oncocytoma and **(E)** papillary renal cell carcinoma, and their respective GAP immunostainings in the right column. **(F)** Immunostaining appears intense in proximal convoluted tubules and is mild to moderate in collecting ducts, **(G)** mild delineating cytoplasmic membranes in clear cell renal cell carcinoma, mild membranous and cytoplasmic in **(H)** chromophobe renal cell carcinoma and **(I)** renal oncocytoma, and **(J)** mild in papillary renal cell carcinoma (GAP immunostaining, haematoxylin counterstaining, original tissue magnification x200).

Figure 4 shows the immunohistochemical results in non-tumour and tumour tissue. The pattern of staining of each tumour subtype coincided fully in every case. GAP immunostaining was strongly positive in proximal convoluted tubules in non-tumour renal tissue and slightly positive in distal tubules. CCRCC showed diffuse and mild membrane immunostaining. PRCC also showed positive immunoreaction located in cytoplasmic membranes, but the pattern of staining was focal. Both ChRCC and RO shared the same immunohistochemical expression with mild and diffuse cytoplasmic staining.

### GAP (*ENPEP*) mRNA levels and CCRCC aggressiveness

Table 3 shows the *ENPEP* mRNA levels in CCRCCs with different Fuhrman’s grade [G1/2 (low) vs. G3/4 (high)] and stage [pT1/2 (organ confined) vs. pT3/4 (non-organ confined)]. *ENPEP* mRNA levels were higher in low grade CCRCCs than in high grade tumours (Table 3a), however, this result was not statistically significant (Mann–Whitney test p = 0.322). Organ confined CCRCCs showed similar ENPEP mRNA levels to non-organ confined tumours (Table 3b) (p = 0.856).

**Table 3:** **GAP (*ENPEP*) mRNA levels in CCRCC**

			
**a**			
	Low Grade (G1-G2)	High Grade (G3-G4)	*P*
*ENPEP*	1065 ± 347	479 ± 138	ns
**b**			
	Low Stage (T1-T2)	High Stage (T3-T4)	*P*
*ENPEP*	707 ± 185	818 ± 383	ns

qRT-PCR data for each analysed sample are recorded as relative units. Values are means ± SE. ns: Not significant (Mann–Whitney test).

## Discussion

Many studies have revealed that peptidases are involved in several physiological functions and play a key role in growth control, differentiation, and signal transduction of several cell systems [31]. Altered expression and catalytic function patterns of these enzymes may contribute to neoplastic transformation and tumour progression [31,32]. Regarding kidney tumours, the altered expression and activity of several peptidases have been evaluated in previous studies [8-10,33-36]. Therefore, the study of peptidase expression and activity appears to be a promising field in the quest for new renal tumour markers and targets. For instance, some studies have led to the design of clinical diagnostic tools, such us neprylisin (NEP/CD10), which is a useful immunohistochemical marker in the diagnosis of proximal nephron-derived carcinomas [33].

In this study we analysed the activity and the expression of a renal cell marker, GAP (or gp160), in a subset of renal tumours and in their non-tumour adjacent tissues. GAP activity of both membrane-bound and soluble fractions was markedly decreased in all tumour subtypes when compared with normal tissues. Interestingly, the decrease in membrane-bound GAP activity revealed a gradient along the different phenotypes of renal neoplasms. Thus, while the activity was twice lower in CCRCC than in normal tissue, the decrease in ChRCC and RO was 16-fold and 34-fold respectively. In addition, we recently demonstrated that this enzyme’s activity decreased 8-fold in PRCC [35]. This finding is similar to some others measuring alternative relevant peptidases, and supports the hypothesis that the loss of several physiologically significant peptidases may be a critical step in the malignant transformation of renal tissues [8,10-12,17,35].

In terms of immunohistochemistry, GAP was strongly expressed in the membrane of proximal tubule cells, whereas tumours from this origin showed only a mild membrane positivity (CCRCC) or even very weak staining (PRCC). This result is in accordance with previous works [8,9] and suggests that the decreases of membrane-bound GAP activity in these neoplasms could be due to loss of protein expression. In addittion, we observed a mild diffuse GAP immunostaining in ChRCC and RO. This pattern is expected for both tumours as they originate from the distal nephron [2]–a specific topography with mild to negative GAP expression, as also shown in other reports [9,15].

Our previous studies demonstrated that several aminopeptidase activities are significantly increased in high stage and high grade CCRCCs [11,12,29,31] and correlated with patient 5-year survival [37], suggesting that these proteases may be a predictor of poor outcome in this disease. In the present work, GAP activity was not found to be correlated with CCRCC patient survival. However, soluble GAP activity significantly increased in high grade (G3-G4) CCRCCs and membrane-bound GAP activity was positively correlated with tumour size, indicating that this peptidase is probably involved in CCRCC growth and aggressiveness.

GAP mRNA levels in renal tumours are similar or even higher than in normal renal tissue [10,11]. In the present work, we observed differences among CCRCCs with low and high Furhman grades, although this result did not reach statistical significance. Similar discrepancies between mRNA expression and enzyme activity or expression have been recently reported in renal carcinomas and in other non-neoplastic kidney diseases when other peptidases were measured [34,38,39]. This finding indicates that these protein modifications could occur at a postranscriptional level and illustrates the importance of assesing protein changes through various means, and not relying solely on mRNA levels [38].

The exact role that GAP plays in renal neoplasms still remains to be clarified. This enzyme has been reported to play a functional role as a regulator of angiotensin II-mediated tumour growth and invasiveness via conversion of locally produced angiotensin II (ang II) in several solid tumours [40]. The downregulation of GAP and other angiotensin-converting peptidases observed in this study and in others [8-11,34] suggests an imbalance in the metabolism of intrarenal angiotensins. However, it is difficult to ascertain which one of these bioactive peptides is more affected in renal neoplasms. We recently showed an important neoexpression of endothelial angiotensin-converting enzyme (ACE) in renal cancer [34], what suggests a higher synthesis of tumour vessel ang II, a vasoactive hormone whose local long-term actions are related to angiogenesis in proliferative disorders [41,42]. Although GAP has been proposed as a functional vascular target in pathologic angiogenic processes [43], this study could not find any GAP neoexpression in renal neoplasm blood vessels. Since this enzyme converts angiotensin II to III, this result could strengthen the hypothesis of an accumulation of ang II in renal tumour vessels, which could stimulate angiogenesis.

GAP has been commonly described as a membrane-bound peptidase, but the activity of soluble isoforms has also been reported in normal and tumour tissues [21-23]. Our data in renal neoplasms have shown distinct patterns of activity in the two subcellular fractions analysed. However, the soluble GAP activity profile was similar to that previously reported with the cytosolic aspartyl-aminopeptidase (EC. 3.4.11.21) in these tumours [10,11]. Therefore, further studies are required to clarify the presence of a soluble isoform of GAP in renal tissues.

In this context, the role of soluble peptidases in the regulation of proliferative diseases is less clear than that of membrane-bound peptidases. Nevertheless, recent findings suggest that a number of peptides and hormones, commonly called “intracrine”, act in the intracellular space after either internalisation or retention in their cells of synthesis [44,45]. Furthermore, intracellular angiotensin II has been reported to also induce cell proliferation in several tissues [44-47]. Therefore, the idea of an intracrine angiotensin dysregulation in kidney tumours should not be ruled out.

## Conclusions

This study demonstrates marked decreases in activity and mild to weak expression of GAP in the four most common histological subtypes of renal neoplasms (CCRCC, PRCC, ChRCC and RO), and a positive correlation between the soluble GAP and CCRCC aggressiveness. These results favour the possibility of a metabolic imbalance among intrarenal angiotensins and a role of GAP in renal neoplastic diseases. A better understanding of the pathophysiological role of GAP in these proliferative disorders will be helpful for designing effective diagnostic, prognostic and therapeutic tools for renal neoplasms.

## Abbreviations

GAP: Glutamyl-aminopeptidase; RCCs: Renal cell carcinomas; CCRCC: Clear cell renal cell carcinoma; PRCC: Papillary renal cell carcinoma; ChRCC: Chromophobe renal cell carcinoma; RO: Renal oncocytoma; UP: Units of peptidase, pmol of product/min.

## Competing interests

All the authors declare no competing interests.

## Authors’ contributions

GL, LB and JIL: research conception and design, edition, revision and approval of the final version. GL, LC and JG: statistical analysis. LB, CES, BS and IP: sample preparation, enzyme assays and protein determinations. FMP, MLC, LB and IP: qRT-PCR assays. JIL: provision of the surgical material and clinical follow-up data of the patients. JIL: selection of appropriate material for immunohistochemical analysis and interpretation of the obtained results. BS and IP: prepared figures and table. GL, LC, LB and JIL: collection and interpretation of final results. All authors read and approved the final manuscript.

## Pre-publication history

The pre-publication history for this paper can be accessed here:

http://www.biomedcentral.com/1471-2407/14/386/prepub
